# Supplementary material for: Metagenomic binning with assembly graph embeddings
Source: Bioinformatics. 2022 Aug 16;38(19):4481–7. doi: 10.1093/bioinformatics/btac557 (PMC9525014; doi:10.1093/bioinformatics/btac557)
Supplement: btac557_supplementary_data [file btac557_supplementary_data.zip › amber outputs.zip!/amber outputs/simHC/genome/vamb_contigs/heatmap_simHC+.pdf]

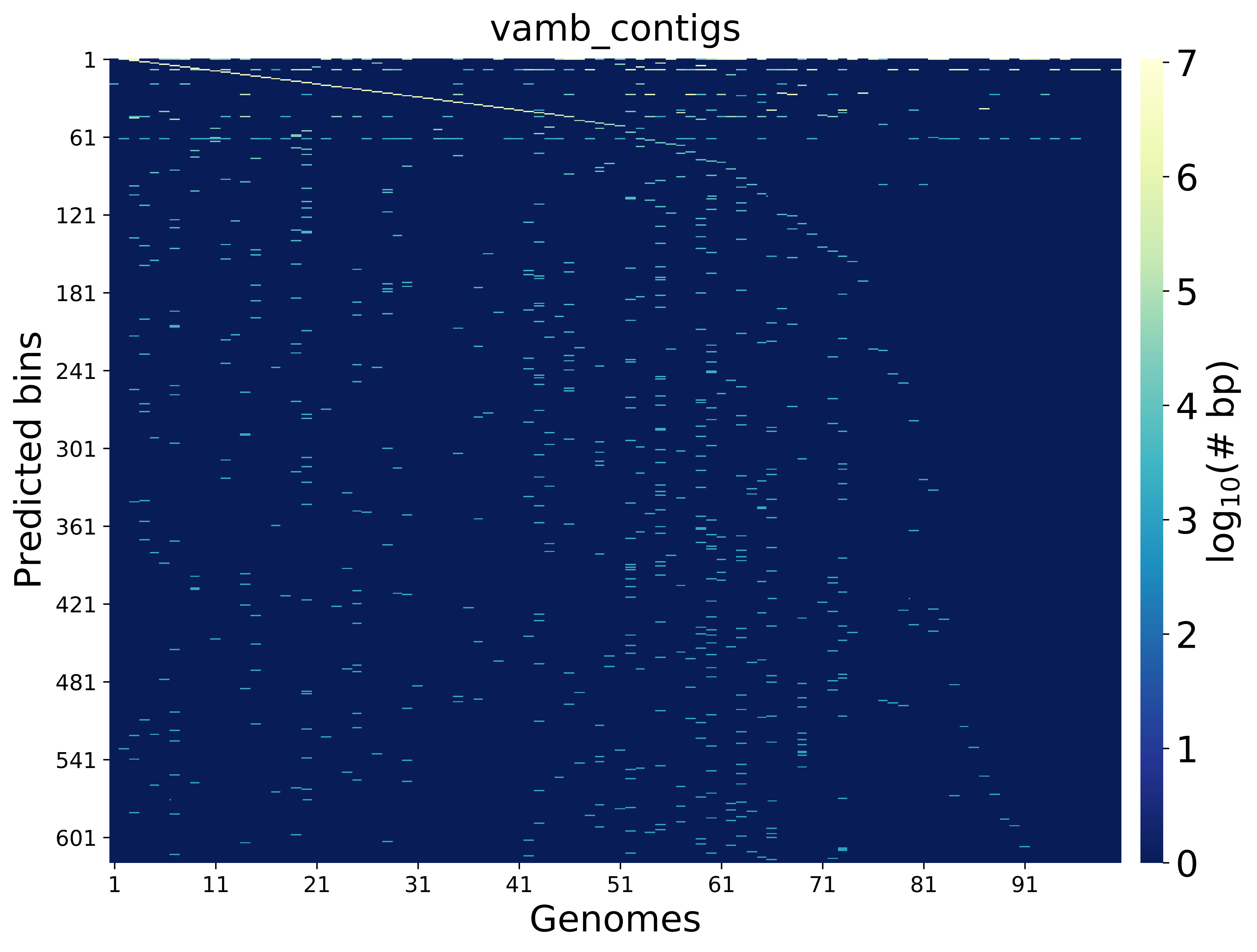

vamb_contigs
Predicted bins
1
61
121
181
241
301
361
421
481
541
601
Genomes
1
11
21
31
41
51
61
71
81
91
log10(# bp)
0
1
2
3
4
5
6
7
